# 2-Ferrocenyl-6-(3-nitro­phen­yl)quinoline

**DOI:** 10.1107/S1600536814004899

**Published:** 2014-03-08

**Authors:** Xiao-Er Yuan, Guo-Qing Shi, Xin Geng, Xin-Qi Hao, Mao-Ping Song

**Affiliations:** a College of Chemistry and Molecular Engineering, Zhengzhou University, Henan, Zhengzhou 450001, People’s Republic of China; b School of Food and Bioengineering, Zhengzhou, University of Light Industry, Henan, Zhengzhou 450052, People’s Republic of China

## Abstract

In the title compound, [Fe(C_5_H_5_)(C_20_H_13_N_2_O_2_)], the substituted cyclo­penta­dienyl ring and quinoline system are approximately coplanar, making a dihedral angle of 5.18 (6)°, while the dihedral angle between the quinoline system and the benzene ring is 28.45 (8)°. There is high thermal motion in the free cyclo­penta­dienyl ring compared with the substituted cyclo­penta­dienyl ring. The conformation of the two cyclopentadienyl rings in the ferrocenyl moiety is eclipsed.

## Related literature   

For ferrocenyl derivatives, see: Staveren & Metzler-Nolte (2004 ▶); Stepnicka (2008 ▶); Xu *et al.* (2010 ▶). For quinolines, see: Carey *et al.* (2006 ▶); Michael (2007 ▶). For the synthesis, see: Xu *et al.* (2013 ▶). 
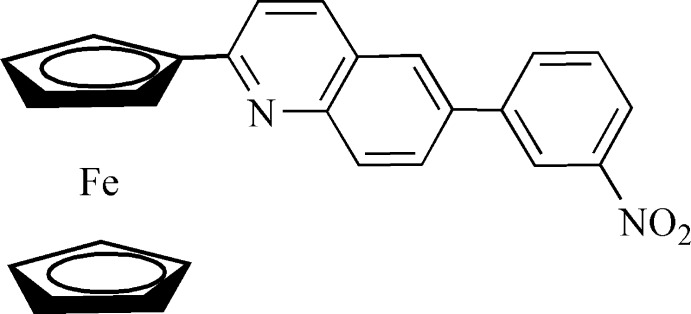


## Experimental   

### 

#### Crystal data  

[Fe(C_5_H_5_)(C_20_H_13_N_2_O_2_)]
*M* *_r_* = 434.26
Monoclinic, 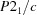

*a* = 12.0523 (15) Å
*b* = 6.6997 (8) Å
*c* = 23.918 (3) Å
β = 91.018 (2)°
*V* = 1931.0 (4) Å^3^
*Z* = 4
Mo *K*α radiation
μ = 0.81 mm^−1^
*T* = 296 K
0.41 × 0.32 × 0.25 mm

#### Data collection  

Bruker SMART APEX CCD area-detector diffractometer
Absorption correction: multi-scan (*SADABS*; Bruker, 2004 ▶) *T*_min_ = 0.733, *T*_max_ = 0.824
10490 measured reflections
3478 independent reflections
2591 reflections with *I* > 2σ(*I*)
*R*_int_ = 0.029

#### Refinement  

*R*[*F* ^2^ > 2σ(*F* ^2^)] = 0.037
*wR*(*F* ^2^) = 0.092
*S* = 1.01
3478 reflections
271 parameters
H-atom parameters constrained
Δρ_max_ = 0.25 e Å^−3^
Δρ_min_ = −0.33 e Å^−3^

### 

Data collection: *APEX2* (Bruker, 2004 ▶); cell refinement: *SAINT* (Bruker, 2004 ▶); data reduction: *SAINT*; program(s) used to solve structure: *SHELXS97* (Sheldrick, 2008 ▶); program(s) used to refine structure: *SHELXL97* (Sheldrick, 2008 ▶); molecular graphics: *SHELXTL* (Sheldrick, 2008 ▶); software used to prepare material for publication: *SHELXL97*.

## Supplementary Material

Crystal structure: contains datablock(s) global, I. DOI: 10.1107/S1600536814004899/fj2663sup1.cif

Structure factors: contains datablock(s) I. DOI: 10.1107/S1600536814004899/fj2663Isup2.hkl

CCDC reference: 989588

Additional supporting information:  crystallographic information; 3D view; checkCIF report

## Figures and Tables

**Fig. 1.**
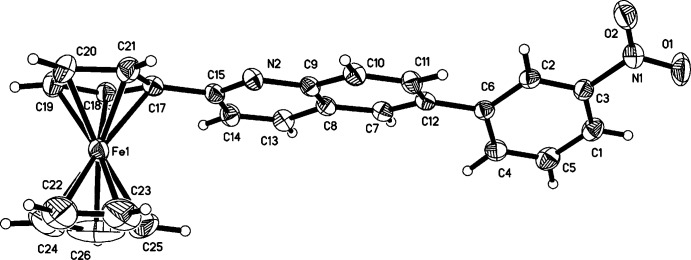
The molecular structure of the title compound with displacement ellipsoids at the 30% probability level.

## supplementary crystallographic information

### 1. Comment

In recent years, there has been an increasing interest in the design of new ferrocenyl derivatives, owing to their utility in diverse fields of chemistry, such as organic synthesis, catalysis and materials science (Staveren & Metzler-Nolte 2004; Stepnicka 2008; Xu *et al.*, 2010). In addition, quinolines and their derivatives are important natural products (Carey *et al.*, 2006; Michael 2007). Here we report the crystal structure of the title compound, derived from the *via A*-alkylation and Suzuki reaction of acetylferrocene, (2-amino-5-bromophenyl)methanol and 3-nitrylphenylboronic acid.

A view on the molecular structure of the title compound is given in Fig. 1. The two cyclopentadienyl rings are almost parallel (dihedral angle of 0.94 (3)°). The substituted cyclopentadienyl and quinolinyl ring are approximately coplanar, making dihedral angle of 5.18 (6)°, and the dihedral angle between the quinolinyl and phenyl ring is 28.45 (8)°.

### 2. Experimental

The title compound was prepared as described in literature (Xu *et al.* 2013) and recrystallized from dichloromethane/petroleum ether solution at room temperature to give the desired crystals suitable for single-crystal X-ray diffraction.

### 3. Refinement

H atoms attached to C atoms of the title compound were placed in geometrically idealized positions and treated as riding with C—H distances constrained to 0.93–0.96 Å, and with *U*ĩso~(H)=1.2Ueq(C).

### Crystal data

**Table:**

[Fe(C_5_H_5_)(C_20_H_13_N_2_O_2_)]	*F*(000) = 896
*M**_r_* = 434.26	*D*_x_ = 1.494 Mg m^−^^3^
Monoclinic, *P*2_1_/*c*	Mo *K*α radiation, λ = 0.71073 Å
Hall symbol: -P 2ybc	Cell parameters from 2663 reflections
*a* = 12.0523 (15) Å	θ = 2.4–24.2°
*b* = 6.6997 (8) Å	µ = 0.81 mm^−^^1^
*c* = 23.918 (3) Å	*T* = 296 K
β = 91.018 (2)°	Block, red
*V* = 1931.0 (4) Å^3^	0.41 × 0.32 × 0.25 mm
*Z* = 4	

### Data collection

**Table:**

Bruker SMART APEX CCD area-detector diffractometer	3478 independent reflections
Radiation source: fine-focus sealed tube	2591 reflections with *I* > 2σ(*I*)
Graphite monochromator	*R*_int_ = 0.029
phi and ω scans	θ_max_ = 25.5°, θ_min_ = 2.4°
Absorption correction: multi-scan (*SADABS*; Bruker, 2004)	*h* = −14→14
*T*_min_ = 0.733, *T*_max_ = 0.824	*k* = −7→7
10490 measured reflections	*l* = −28→28

### Refinement

**Table:**

Refinement on *F*^2^	Primary atom site location: structure-invariant direct methods
Least-squares matrix: full	Secondary atom site location: difference Fourier map
*R*[*F*^2^ > 2σ(*F*^2^)] = 0.037	Hydrogen site location: inferred from neighbouring sites
*wR*(*F*^2^) = 0.092	H-atom parameters constrained
*S* = 1.01	*w* = 1/[σ^2^(*F*_o_^2^) + (0.0347*P*)^2^ + 1.1805*P*] where *P* = (*F*_o_^2^ + 2*F*_c_^2^)/3
3478 reflections	(Δ/σ)_max_ = 0.018
271 parameters	Δρ_max_ = 0.25 e Å^−^^3^
0 restraints	Δρ_min_ = −0.33 e Å^−^^3^

### Special details

**Table:**

Geometry. All e.s.d.'s (except the e.s.d. in the dihedral angle between two l.s. planes)are estimated using the full covariance matrix. The cell e.s.d.'s are takeninto account individually in the estimation of e.s.d.'s in distances, anglesand torsion angles; correlations between e.s.d.'s in cell parameters are onlyused when they are defined by crystal symmetry. An approximate (isotropic)treatment of cell e.s.d.'s is used for estimating e.s.d.'s involving l.s. planes.
Refinement. Refinement of *F*^2^ against ALL reflections. The weighted *R*-factor *wR* and goodness of fit *S* are based on *F*^2^, conventional *R*-factors *R* are based on *F*, with *F* set to zero for negative *F*^2^. The threshold expression of *F*^2^ > σ(*F*^2^) is used only for calculating *R*-factors(gt) *etc*. and is not relevant to the choice of reflections for refinement. *R*-factors based on *F*^2^ are statistically about twice as large as those based on *F*, and *R*- factors based on ALL data will be even larger.

### Fractional atomic coordinates and isotropic or equivalent isotropic displacement parameters (Å^2^)

**Table:**

	*x*	*y*	*z*	*U*_iso_*/*U*_eq_	
Fe1	0.48569 (3)	0.34296 (6)	0.382218 (16)	0.04797 (15)	
N1	1.19484 (19)	0.4758 (4)	0.00580 (10)	0.0552 (6)	
N2	0.75102 (16)	0.3060 (3)	0.29398 (9)	0.0424 (5)	
O1	1.2363 (2)	0.5509 (4)	−0.03524 (9)	0.0823 (7)	
O2	1.2073 (2)	0.3023 (4)	0.01888 (10)	0.0812 (7)	
C1	1.1192 (2)	0.8036 (4)	0.02870 (12)	0.0511 (7)	
H1	1.1580	0.8580	−0.0009	0.061*	
C2	1.07022 (19)	0.5188 (4)	0.08508 (10)	0.0407 (6)	
H2	1.0780	0.3831	0.0925	0.049*	
C3	1.1253 (2)	0.6038 (4)	0.04103 (10)	0.0417 (6)	
C4	0.9954 (2)	0.8381 (4)	0.10550 (11)	0.0496 (7)	
H4	0.9501	0.9195	0.1268	0.060*	
C5	1.0530 (2)	0.9205 (4)	0.06205 (12)	0.0565 (8)	
H5	1.0473	1.0567	0.0550	0.068*	
C6	1.00316 (19)	0.6358 (4)	0.11839 (10)	0.0381 (6)	
C7	0.91613 (19)	0.6649 (4)	0.21141 (10)	0.0399 (6)	
H7	0.9433	0.7946	0.2138	0.048*	
C8	0.85097 (19)	0.5899 (4)	0.25508 (10)	0.0371 (6)	
C9	0.81385 (18)	0.3900 (4)	0.25303 (10)	0.0374 (6)	
C10	0.8399 (2)	0.2741 (4)	0.20579 (11)	0.0429 (6)	
H10	0.8158	0.1425	0.2035	0.051*	
C11	0.9000 (2)	0.3533 (4)	0.16343 (11)	0.0427 (6)	
H11	0.9148	0.2749	0.1324	0.051*	
C12	0.94044 (18)	0.5517 (4)	0.16546 (10)	0.0375 (6)	
C13	0.8167 (2)	0.7074 (4)	0.30100 (11)	0.0446 (6)	
H13	0.8384	0.8404	0.3039	0.053*	
C14	0.7524 (2)	0.6244 (4)	0.34049 (11)	0.0462 (7)	
H14	0.7281	0.7008	0.3703	0.055*	
C15	0.72202 (19)	0.4201 (4)	0.33620 (10)	0.0407 (6)	
C17	0.65424 (19)	0.3269 (4)	0.37962 (11)	0.0438 (6)	
C18	0.6198 (2)	0.4143 (5)	0.43104 (11)	0.0515 (7)	
H18	0.6393	0.5482	0.4445	0.062*	
C19	0.5526 (2)	0.2750 (5)	0.45884 (12)	0.0619 (8)	
H19	0.5170	0.2955	0.4949	0.074*	
C20	0.5448 (3)	0.1018 (5)	0.42547 (13)	0.0670 (9)	
H20	0.5026	−0.0188	0.4344	0.080*	
C21	0.6071 (2)	0.1327 (4)	0.37660 (12)	0.0559 (8)	
H21	0.6158	0.0373	0.3459	0.067*	
C22	0.3303 (4)	0.4402 (15)	0.3922 (2)	0.126 (2)	
H22	0.2929	0.4505	0.4281	0.151*	
C23	0.3859 (5)	0.3224 (11)	0.3142 (2)	0.1147 (18)	
H23	0.3954	0.2306	0.2827	0.138*	
C24	0.3271 (3)	0.2868 (10)	0.3600 (3)	0.1131 (18)	
H24	0.2872	0.1627	0.3677	0.136*	
C25	0.4280 (3)	0.5081 (14)	0.3183 (3)	0.135 (3)	
H25	0.4724	0.5773	0.2904	0.162*	
C26	0.3912 (6)	0.5869 (8)	0.3687 (4)	0.147 (3)	
H26	0.4040	0.7218	0.3833	0.176*	

### Atomic displacement parameters (Å^2^)

**Table:**

	*U*^11^	*U*^22^	*U*^33^	*U*^12^	*U*^13^	*U*^23^
Fe1	0.0360 (2)	0.0635 (3)	0.0444 (2)	−0.00707 (19)	0.00264 (16)	0.0053 (2)
N1	0.0543 (14)	0.0625 (18)	0.0490 (14)	0.0005 (13)	0.0113 (11)	0.0034 (13)
N2	0.0354 (11)	0.0452 (13)	0.0466 (13)	−0.0026 (10)	0.0048 (10)	−0.0012 (10)
O1	0.1055 (18)	0.0832 (17)	0.0598 (14)	0.0021 (14)	0.0434 (13)	0.0112 (13)
O2	0.1048 (18)	0.0609 (16)	0.0795 (16)	0.0234 (13)	0.0436 (14)	0.0120 (13)
C1	0.0530 (16)	0.0513 (19)	0.0490 (16)	−0.0093 (14)	0.0012 (13)	0.0103 (14)
C2	0.0420 (14)	0.0371 (15)	0.0431 (14)	0.0007 (11)	−0.0010 (11)	0.0036 (12)
C3	0.0395 (14)	0.0480 (17)	0.0376 (14)	−0.0018 (12)	0.0005 (11)	0.0010 (12)
C4	0.0537 (16)	0.0427 (17)	0.0525 (17)	0.0030 (13)	0.0029 (13)	0.0015 (14)
C5	0.0687 (19)	0.0399 (17)	0.0608 (18)	−0.0014 (14)	0.0010 (15)	0.0088 (14)
C6	0.0359 (13)	0.0386 (15)	0.0397 (13)	−0.0016 (11)	−0.0027 (10)	0.0012 (12)
C7	0.0354 (13)	0.0360 (15)	0.0483 (15)	−0.0029 (11)	0.0000 (11)	−0.0017 (12)
C8	0.0301 (12)	0.0392 (15)	0.0419 (14)	−0.0019 (10)	−0.0017 (10)	−0.0040 (11)
C9	0.0292 (12)	0.0414 (16)	0.0414 (14)	−0.0010 (10)	0.0002 (10)	−0.0017 (11)
C10	0.0439 (14)	0.0329 (15)	0.0520 (16)	−0.0011 (11)	0.0048 (12)	−0.0036 (12)
C11	0.0450 (14)	0.0390 (16)	0.0441 (14)	0.0012 (12)	0.0065 (12)	−0.0069 (12)
C12	0.0314 (12)	0.0400 (15)	0.0411 (14)	0.0027 (11)	−0.0008 (10)	0.0004 (12)
C13	0.0458 (15)	0.0413 (16)	0.0466 (15)	−0.0040 (12)	−0.0003 (12)	−0.0062 (12)
C14	0.0456 (15)	0.0512 (18)	0.0419 (15)	−0.0011 (13)	0.0043 (12)	−0.0073 (13)
C15	0.0305 (12)	0.0496 (17)	0.0421 (14)	0.0006 (11)	0.0007 (11)	0.0001 (12)
C17	0.0336 (13)	0.0534 (18)	0.0444 (15)	0.0022 (12)	−0.0002 (11)	0.0034 (13)
C18	0.0418 (15)	0.070 (2)	0.0424 (15)	−0.0027 (14)	0.0003 (12)	0.0001 (14)
C19	0.0558 (18)	0.084 (2)	0.0461 (17)	−0.0022 (17)	0.0056 (14)	0.0113 (17)
C20	0.072 (2)	0.065 (2)	0.064 (2)	−0.0063 (17)	0.0115 (17)	0.0228 (17)
C21	0.0610 (18)	0.0496 (19)	0.0574 (18)	−0.0014 (14)	0.0090 (14)	0.0073 (14)
C22	0.065 (3)	0.235 (8)	0.078 (3)	0.065 (4)	−0.002 (2)	−0.016 (4)
C23	0.090 (3)	0.187 (6)	0.066 (3)	0.032 (4)	−0.030 (3)	−0.032 (3)
C24	0.052 (2)	0.148 (5)	0.139 (5)	−0.037 (3)	−0.030 (3)	0.041 (4)
C25	0.047 (2)	0.217 (7)	0.140 (5)	−0.007 (4)	−0.012 (3)	0.126 (5)
C26	0.122 (5)	0.073 (3)	0.241 (9)	0.029 (3)	−0.112 (6)	−0.017 (4)

### Geometric parameters (Å, º)

**Table:**

Fe1—C22	2.001 (4)	C8—C13	1.419 (3)
Fe1—C25	2.001 (4)	C9—C10	1.411 (3)
Fe1—C24	2.010 (4)	C10—C11	1.364 (3)
Fe1—C23	2.011 (4)	C10—H10	0.9300
Fe1—C26	2.015 (5)	C11—C12	1.416 (3)
Fe1—C18	2.035 (3)	C11—H11	0.9300
Fe1—C21	2.037 (3)	C13—C14	1.353 (3)
Fe1—C17	2.036 (2)	C13—H13	0.9300
Fe1—C20	2.040 (3)	C14—C15	1.420 (4)
Fe1—C19	2.041 (3)	C14—H14	0.9300
N1—O2	1.213 (3)	C15—C17	1.472 (3)
N1—O1	1.218 (3)	C17—C21	1.421 (4)
N1—C3	1.474 (3)	C17—C18	1.430 (4)
N2—C15	1.319 (3)	C18—C19	1.410 (4)
N2—C9	1.370 (3)	C18—H18	0.9800
C1—C3	1.372 (4)	C19—C20	1.411 (5)
C1—C5	1.382 (4)	C19—H19	0.9800
C1—H1	0.9300	C20—C21	1.416 (4)
C2—C3	1.378 (3)	C20—H20	0.9800
C2—C6	1.388 (3)	C21—H21	0.9800
C2—H2	0.9300	C22—C24	1.284 (7)
C4—C5	1.376 (4)	C22—C26	1.354 (8)
C4—C6	1.393 (3)	C22—H22	0.9800
C4—H4	0.9300	C23—C24	1.337 (7)
C5—H5	0.9300	C23—C25	1.347 (8)
C6—C12	1.479 (3)	C23—H23	0.9800
C7—C12	1.372 (3)	C24—H24	0.9800
C7—C8	1.410 (3)	C25—C26	1.396 (8)
C7—H7	0.9300	C25—H25	0.9800
C8—C9	1.413 (3)	C26—H26	0.9800
			
C22—Fe1—C25	66.3 (2)	C11—C10—H10	119.7
C22—Fe1—C24	37.3 (2)	C9—C10—H10	119.7
C25—Fe1—C24	65.5 (2)	C10—C11—C12	121.8 (2)
C22—Fe1—C23	64.61 (19)	C10—C11—H11	119.1
C25—Fe1—C23	39.2 (2)	C12—C11—H11	119.1
C24—Fe1—C23	38.8 (2)	C7—C12—C11	117.9 (2)
C22—Fe1—C26	39.4 (2)	C7—C12—C6	121.2 (2)
C25—Fe1—C26	40.7 (2)	C11—C12—C6	120.8 (2)
C24—Fe1—C26	65.0 (2)	C14—C13—C8	119.4 (2)
C23—Fe1—C26	66.3 (2)	C14—C13—H13	120.3
C22—Fe1—C18	126.2 (2)	C8—C13—H13	120.3
C25—Fe1—C18	124.8 (2)	C13—C14—C15	119.8 (2)
C24—Fe1—C18	159.7 (2)	C13—C14—H14	120.1
C23—Fe1—C18	159.8 (2)	C15—C14—H14	120.1
C26—Fe1—C18	110.03 (17)	N2—C15—C14	122.9 (2)
C22—Fe1—C21	155.2 (3)	N2—C15—C17	116.9 (2)
C25—Fe1—C21	124.9 (3)	C14—C15—C17	120.2 (2)
C24—Fe1—C21	122.3 (2)	C21—C17—C18	107.3 (2)
C23—Fe1—C21	108.60 (17)	C21—C17—C15	125.4 (2)
C26—Fe1—C21	162.7 (3)	C18—C17—C15	127.3 (3)
C18—Fe1—C21	68.65 (12)	C21—C17—Fe1	69.60 (15)
C22—Fe1—C17	163.1 (3)	C18—C17—Fe1	69.37 (14)
C25—Fe1—C17	109.82 (14)	C15—C17—Fe1	124.45 (18)
C24—Fe1—C17	157.9 (2)	C19—C18—C17	108.2 (3)
C23—Fe1—C17	123.65 (18)	C19—C18—Fe1	69.99 (17)
C26—Fe1—C17	126.8 (3)	C17—C18—Fe1	69.50 (15)
C18—Fe1—C17	41.13 (10)	C19—C18—H18	125.9
C21—Fe1—C17	40.84 (11)	C17—C18—H18	125.9
C22—Fe1—C20	121.1 (3)	Fe1—C18—H18	125.9
C25—Fe1—C20	159.8 (3)	C20—C19—C18	108.1 (3)
C24—Fe1—C20	107.92 (18)	C20—C19—Fe1	69.75 (18)
C23—Fe1—C20	123.7 (2)	C18—C19—Fe1	69.53 (16)
C26—Fe1—C20	156.2 (3)	C20—C19—H19	125.9
C18—Fe1—C20	68.18 (13)	C18—C19—H19	125.9
C21—Fe1—C20	40.64 (11)	Fe1—C19—H19	125.9
C17—Fe1—C20	68.55 (12)	C19—C20—C21	108.4 (3)
C22—Fe1—C19	108.77 (16)	C19—C20—Fe1	69.79 (18)
C25—Fe1—C19	159.3 (3)	C21—C20—Fe1	69.56 (17)
C24—Fe1—C19	123.70 (19)	C19—C20—H20	125.8
C23—Fe1—C19	158.9 (3)	C21—C20—H20	125.8
C26—Fe1—C19	122.6 (3)	Fe1—C20—H20	125.8
C18—Fe1—C19	40.48 (11)	C20—C21—C17	108.0 (3)
C21—Fe1—C19	68.41 (13)	C20—C21—Fe1	69.81 (18)
C17—Fe1—C19	68.71 (11)	C17—C21—Fe1	69.56 (15)
C20—Fe1—C19	40.45 (13)	C20—C21—H21	126.0
O2—N1—O1	123.6 (3)	C17—C21—H21	126.0
O2—N1—C3	118.6 (2)	Fe1—C21—H21	126.0
O1—N1—C3	117.8 (3)	C24—C22—C26	110.1 (5)
C15—N2—C9	117.8 (2)	C24—C22—Fe1	71.7 (3)
C3—C1—C5	117.3 (3)	C26—C22—Fe1	70.9 (3)
C3—C1—H1	121.4	C24—C22—H22	125.0
C5—C1—H1	121.4	C26—C22—H22	124.9
C3—C2—C6	119.9 (2)	Fe1—C22—H22	125.0
C3—C2—H2	120.0	C24—C23—C25	108.0 (5)
C6—C2—H2	120.0	C24—C23—Fe1	70.5 (2)
C1—C3—C2	122.9 (2)	C25—C23—Fe1	70.0 (3)
C1—C3—N1	118.3 (2)	C24—C23—H23	126.0
C2—C3—N1	118.9 (2)	C25—C23—H23	126.0
C5—C4—C6	121.6 (3)	Fe1—C23—H23	126.0
C5—C4—H4	119.2	C22—C24—C23	109.8 (5)
C6—C4—H4	119.2	C22—C24—Fe1	70.9 (3)
C4—C5—C1	120.9 (3)	C23—C24—Fe1	70.6 (2)
C4—C5—H5	119.6	C22—C24—H24	125.1
C1—C5—H5	119.6	C23—C24—H24	125.1
C2—C6—C4	117.4 (2)	Fe1—C24—H24	125.1
C2—C6—C12	122.2 (2)	C23—C25—C26	106.8 (5)
C4—C6—C12	120.4 (2)	C23—C25—Fe1	70.8 (3)
C12—C7—C8	121.7 (2)	C26—C25—Fe1	70.2 (3)
C12—C7—H7	119.1	C23—C25—H25	126.6
C8—C7—H7	119.1	C26—C25—H25	126.6
C7—C8—C9	119.5 (2)	Fe1—C25—H25	126.6
C7—C8—C13	123.3 (2)	C22—C26—C25	105.4 (5)
C9—C8—C13	117.2 (2)	C22—C26—Fe1	69.7 (3)
N2—C9—C10	118.7 (2)	C25—C26—Fe1	69.1 (3)
N2—C9—C8	122.9 (2)	C22—C26—H26	127.3
C10—C9—C8	118.4 (2)	C25—C26—H26	127.3
C11—C10—C9	120.6 (2)	Fe1—C26—H26	127.3
			
C5—C1—C3—C2	−1.0 (4)	C23—Fe1—C20—C21	79.2 (3)
C5—C1—C3—N1	179.6 (2)	C26—Fe1—C20—C21	−172.6 (4)
C6—C2—C3—C1	1.4 (4)	C18—Fe1—C20—C21	−82.20 (19)
C6—C2—C3—N1	−179.2 (2)	C17—Fe1—C20—C21	−37.79 (18)
O2—N1—C3—C1	174.6 (3)	C19—Fe1—C20—C21	−119.7 (3)
O1—N1—C3—C1	−5.8 (4)	C19—C20—C21—C17	0.1 (4)
O2—N1—C3—C2	−4.8 (4)	Fe1—C20—C21—C17	59.3 (2)
O1—N1—C3—C2	174.8 (2)	C19—C20—C21—Fe1	−59.2 (2)
C6—C4—C5—C1	1.4 (4)	C18—C17—C21—C20	0.0 (3)
C3—C1—C5—C4	−0.4 (4)	C15—C17—C21—C20	−177.8 (2)
C3—C2—C6—C4	−0.4 (4)	Fe1—C17—C21—C20	−59.4 (2)
C3—C2—C6—C12	178.8 (2)	C18—C17—C21—Fe1	59.39 (18)
C5—C4—C6—C2	−1.0 (4)	C15—C17—C21—Fe1	−118.4 (2)
C5—C4—C6—C12	179.8 (2)	C22—Fe1—C21—C20	−50.9 (5)
C12—C7—C8—C9	3.1 (4)	C25—Fe1—C21—C20	−160.8 (3)
C12—C7—C8—C13	−174.8 (2)	C24—Fe1—C21—C20	−79.8 (3)
C15—N2—C9—C10	−176.1 (2)	C23—Fe1—C21—C20	−120.4 (3)
C15—N2—C9—C8	1.9 (3)	C26—Fe1—C21—C20	169.9 (6)
C7—C8—C9—N2	179.5 (2)	C18—Fe1—C21—C20	81.0 (2)
C13—C8—C9—N2	−2.5 (4)	C17—Fe1—C21—C20	119.3 (3)
C7—C8—C9—C10	−2.5 (3)	C19—Fe1—C21—C20	37.30 (19)
C13—C8—C9—C10	175.4 (2)	C22—Fe1—C21—C17	−170.2 (4)
N2—C9—C10—C11	178.5 (2)	C25—Fe1—C21—C17	79.9 (3)
C8—C9—C10—C11	0.4 (4)	C24—Fe1—C21—C17	160.9 (2)
C9—C10—C11—C12	1.3 (4)	C23—Fe1—C21—C17	120.3 (3)
C8—C7—C12—C11	−1.4 (4)	C26—Fe1—C21—C17	50.6 (6)
C8—C7—C12—C6	176.3 (2)	C18—Fe1—C21—C17	−38.33 (16)
C10—C11—C12—C7	−0.8 (4)	C20—Fe1—C21—C17	−119.3 (3)
C10—C11—C12—C6	−178.5 (2)	C19—Fe1—C21—C17	−81.99 (18)
C2—C6—C12—C7	150.7 (2)	C25—Fe1—C22—C24	80.2 (4)
C4—C6—C12—C7	−30.1 (4)	C23—Fe1—C22—C24	36.9 (4)
C2—C6—C12—C11	−31.7 (3)	C26—Fe1—C22—C24	119.8 (5)
C4—C6—C12—C11	147.5 (3)	C18—Fe1—C22—C24	−163.1 (3)
C7—C8—C13—C14	178.7 (2)	C21—Fe1—C22—C24	−42.4 (6)
C9—C8—C13—C14	0.8 (4)	C17—Fe1—C22—C24	160.2 (4)
C8—C13—C14—C15	1.4 (4)	C20—Fe1—C22—C24	−78.6 (4)
C9—N2—C15—C14	0.5 (4)	C19—Fe1—C22—C24	−121.4 (4)
C9—N2—C15—C17	179.8 (2)	C25—Fe1—C22—C26	−39.6 (4)
C13—C14—C15—N2	−2.1 (4)	C24—Fe1—C22—C26	−119.8 (5)
C13—C14—C15—C17	178.5 (2)	C23—Fe1—C22—C26	−82.9 (4)
N2—C15—C17—C21	−7.7 (4)	C18—Fe1—C22—C26	77.1 (5)
C14—C15—C17—C21	171.6 (3)	C21—Fe1—C22—C26	−162.2 (4)
N2—C15—C17—C18	174.9 (2)	C17—Fe1—C22—C26	40.4 (8)
C14—C15—C17—C18	−5.8 (4)	C20—Fe1—C22—C26	161.6 (4)
N2—C15—C17—Fe1	−96.0 (3)	C19—Fe1—C22—C26	118.8 (4)
C14—C15—C17—Fe1	83.4 (3)	C22—Fe1—C23—C24	−35.5 (4)
C22—Fe1—C17—C21	165.7 (6)	C25—Fe1—C23—C24	−118.5 (5)
C25—Fe1—C17—C21	−120.9 (4)	C26—Fe1—C23—C24	−79.0 (4)
C24—Fe1—C17—C21	−47.3 (5)	C18—Fe1—C23—C24	−162.7 (5)
C23—Fe1—C17—C21	−79.4 (3)	C21—Fe1—C23—C24	118.7 (4)
C26—Fe1—C17—C21	−163.4 (4)	C17—Fe1—C23—C24	161.4 (4)
C18—Fe1—C17—C21	118.6 (2)	C20—Fe1—C23—C24	76.2 (4)
C20—Fe1—C17—C21	37.61 (18)	C19—Fe1—C23—C24	40.6 (7)
C19—Fe1—C17—C21	81.20 (19)	C22—Fe1—C23—C25	82.9 (4)
C22—Fe1—C17—C18	47.2 (6)	C24—Fe1—C23—C25	118.5 (5)
C25—Fe1—C17—C18	120.6 (4)	C26—Fe1—C23—C25	39.5 (3)
C24—Fe1—C17—C18	−165.9 (4)	C18—Fe1—C23—C25	−44.2 (7)
C23—Fe1—C17—C18	162.0 (3)	C21—Fe1—C23—C25	−122.8 (4)
C26—Fe1—C17—C18	78.1 (4)	C17—Fe1—C23—C25	−80.1 (4)
C21—Fe1—C17—C18	−118.6 (2)	C20—Fe1—C23—C25	−165.3 (4)
C20—Fe1—C17—C18	−80.96 (19)	C19—Fe1—C23—C25	159.1 (5)
C19—Fe1—C17—C18	−37.37 (18)	C26—C22—C24—C23	0.6 (6)
C22—Fe1—C17—C15	−74.7 (6)	Fe1—C22—C24—C23	−60.2 (3)
C25—Fe1—C17—C15	−1.3 (4)	C26—C22—C24—Fe1	60.8 (4)
C24—Fe1—C17—C15	72.3 (5)	C25—C23—C24—C22	0.1 (6)
C23—Fe1—C17—C15	40.2 (4)	Fe1—C23—C24—C22	60.4 (4)
C26—Fe1—C17—C15	−43.7 (4)	C25—C23—C24—Fe1	−60.2 (3)
C18—Fe1—C17—C15	−121.8 (3)	C25—Fe1—C24—C22	−82.4 (4)
C21—Fe1—C17—C15	119.6 (3)	C23—Fe1—C24—C22	−120.0 (5)
C20—Fe1—C17—C15	157.2 (3)	C26—Fe1—C24—C22	−37.4 (4)
C19—Fe1—C17—C15	−159.2 (3)	C18—Fe1—C24—C22	42.7 (7)
C21—C17—C18—C19	0.0 (3)	C21—Fe1—C24—C22	160.5 (4)
C15—C17—C18—C19	177.7 (2)	C17—Fe1—C24—C22	−164.9 (4)
Fe1—C17—C18—C19	59.50 (19)	C20—Fe1—C24—C22	118.1 (4)
C21—C17—C18—Fe1	−59.53 (19)	C19—Fe1—C24—C22	76.3 (4)
C15—C17—C18—Fe1	118.2 (3)	C22—Fe1—C24—C23	120.0 (5)
C22—Fe1—C18—C19	75.9 (4)	C25—Fe1—C24—C23	37.6 (4)
C25—Fe1—C18—C19	160.2 (3)	C26—Fe1—C24—C23	82.6 (4)
C24—Fe1—C18—C19	45.2 (6)	C18—Fe1—C24—C23	162.7 (5)
C23—Fe1—C18—C19	−167.3 (5)	C21—Fe1—C24—C23	−79.5 (4)
C26—Fe1—C18—C19	117.1 (4)	C17—Fe1—C24—C23	−44.9 (7)
C21—Fe1—C18—C19	−81.3 (2)	C20—Fe1—C24—C23	−121.8 (4)
C17—Fe1—C18—C19	−119.4 (3)	C19—Fe1—C24—C23	−163.6 (4)
C20—Fe1—C18—C19	−37.49 (19)	C24—C23—C25—C26	−0.8 (5)
C22—Fe1—C18—C17	−164.7 (3)	Fe1—C23—C25—C26	−61.4 (3)
C25—Fe1—C18—C17	−80.4 (4)	C24—C23—C25—Fe1	60.6 (3)
C24—Fe1—C18—C17	164.6 (5)	C22—Fe1—C25—C23	−78.3 (4)
C23—Fe1—C18—C17	−47.9 (6)	C24—Fe1—C25—C23	−37.3 (3)
C26—Fe1—C18—C17	−123.5 (4)	C26—Fe1—C25—C23	−116.7 (5)
C21—Fe1—C18—C17	38.07 (16)	C18—Fe1—C25—C23	162.9 (3)
C20—Fe1—C18—C17	81.92 (18)	C21—Fe1—C25—C23	76.2 (3)
C19—Fe1—C18—C17	119.4 (3)	C17—Fe1—C25—C23	119.3 (3)
C17—C18—C19—C20	0.1 (3)	C20—Fe1—C25—C23	37.8 (6)
Fe1—C18—C19—C20	59.3 (2)	C19—Fe1—C25—C23	−158.6 (4)
C17—C18—C19—Fe1	−59.20 (18)	C22—Fe1—C25—C26	38.4 (3)
C22—Fe1—C19—C20	116.3 (4)	C24—Fe1—C25—C26	79.5 (4)
C25—Fe1—C19—C20	−171.4 (4)	C23—Fe1—C25—C26	116.7 (5)
C24—Fe1—C19—C20	77.8 (3)	C18—Fe1—C25—C26	−80.3 (4)
C23—Fe1—C19—C20	48.4 (5)	C21—Fe1—C25—C26	−167.1 (4)
C26—Fe1—C19—C20	157.6 (4)	C17—Fe1—C25—C26	−123.9 (4)
C18—Fe1—C19—C20	−119.4 (3)	C20—Fe1—C25—C26	154.5 (5)
C21—Fe1—C19—C20	−37.47 (18)	C19—Fe1—C25—C26	−41.9 (6)
C17—Fe1—C19—C20	−81.50 (19)	C24—C22—C26—C25	−1.0 (6)
C22—Fe1—C19—C18	−124.3 (4)	Fe1—C22—C26—C25	60.3 (3)
C25—Fe1—C19—C18	−51.9 (5)	C24—C22—C26—Fe1	−61.3 (4)
C24—Fe1—C19—C18	−162.8 (3)	C23—C25—C26—C22	1.1 (5)
C23—Fe1—C19—C18	167.8 (4)	Fe1—C25—C26—C22	−60.7 (3)
C26—Fe1—C19—C18	−83.0 (4)	C23—C25—C26—Fe1	61.8 (3)
C21—Fe1—C19—C18	81.98 (19)	C25—Fe1—C26—C22	116.4 (5)
C17—Fe1—C19—C18	37.95 (17)	C24—Fe1—C26—C22	35.5 (3)
C20—Fe1—C19—C18	119.4 (3)	C23—Fe1—C26—C22	78.3 (4)
C18—C19—C20—C21	−0.1 (4)	C18—Fe1—C26—C22	−123.2 (4)
Fe1—C19—C20—C21	59.0 (2)	C21—Fe1—C26—C22	154.4 (5)
C18—C19—C20—Fe1	−59.1 (2)	C17—Fe1—C26—C22	−166.4 (3)
C22—Fe1—C20—C19	−82.6 (3)	C20—Fe1—C26—C22	−42.0 (7)
C25—Fe1—C20—C19	171.2 (4)	C19—Fe1—C26—C22	−79.9 (4)
C24—Fe1—C20—C19	−121.3 (3)	C22—Fe1—C26—C25	−116.4 (5)
C23—Fe1—C20—C19	−161.1 (2)	C24—Fe1—C26—C25	−80.9 (4)
C26—Fe1—C20—C19	−52.9 (5)	C23—Fe1—C26—C25	−38.1 (3)
C18—Fe1—C20—C19	37.52 (17)	C18—Fe1—C26—C25	120.4 (4)
C21—Fe1—C20—C19	119.7 (3)	C21—Fe1—C26—C25	38.1 (8)
C17—Fe1—C20—C19	81.92 (19)	C17—Fe1—C26—C25	77.2 (4)
C22—Fe1—C20—C21	157.7 (3)	C20—Fe1—C26—C25	−158.4 (5)
C25—Fe1—C20—C21	51.5 (5)	C19—Fe1—C26—C25	163.7 (4)
C24—Fe1—C20—C21	119.0 (3)		
